# Cleavage-stage or blastocyst-stage embryo biopsy has no impact on growth and health in children up to 2 years of age

**DOI:** 10.1186/s12958-023-01140-3

**Published:** 2023-09-22

**Authors:** Florence Belva, Fiskani Kondowe, Anick De Vos, Kathelijn Keymolen, Andrea Buysse, Frederik Hes, Veerle Berckmoes, Pieter Verdyck, Willem Verpoest, Martine De Rycke

**Affiliations:** 1 Vrije Universiteit Brussel (VUB), Universitair Ziekenhuis Brussel (UZ Brussel), Clinical Sciences, Research Group Reproduction and Genetics, Centre for Medical Genetics, Brussels, Belgium; 2 Centre for Biostatistics, Division of Population Health, Health Services Research & Primary Care, School of Health Sciences, Faculty of Biology, Medicine and Health, The University of Manchester, Manchester Academic Health Science Centre, Manchester, UK; 3 Vrije Universiteit Brussel (VUB), Universitair Ziekenhuis Brussel (UZ Brussel), Clinical Sciences, Research Group Reproduction and Genetics, Brussels IVF Centre for Reproductive Medicine, Brussels, Belgium

**Keywords:** Embryo biopsy, Health, Children, Cleavage, Blastocyst, PGT

## Abstract

**Background:**

Studies show conflicting results on neonatal outcomes following embryo biopsy for PGT, primarily due to small sample sizes and/or heterogeneity in the timing of embryo biopsy (day 3; EBD3 or day 5/6; EBD5) and type of embryo transfer. Even fewer data exist on the impact on children’s health beyond the neonatal period. This study aimed to explore outcomes in children born after EBD3 or EBD5 followed by fresh (FRESH) or frozen-thawed embryo transfer (FET).

**Methods:**

This single-centre cohort study compared birth data of 630 children after EBD3, of 222 EBD5 and of 1532 after non-biopsied embryo transfers performed between 2014 and 2018. Follow-up data on growth were available for 426, 131 and 662 children, respectively.

**Results:**

Embryo biopsy, either at EBD3 or EBD5 in FET and FRESH cycles did not negatively affect anthropometry at birth, infancy or childhood compared to outcomes in non-biopsied FET and FRESH cycles.

While there was no adverse effect of the timing of embryo biopsy (EBD3 versus EBD5), children born after EBD3 followed by FET had larger sizes at birth, but not thereafter, than children born after EBD3 followed by FRESH.

Reassuringly, weight and height gain, proportions of major congenital malformations, developmental problems, hospital admissions and surgical interventions were similar between comparison groups.

**Conclusion:**

Our study indicated that neither EBD3 nor EBD5 followed by FRESH or FET had a negative impact on anthropometry and on health outcomes up to 2 years of age.

**Supplementary Information:**

The online version contains supplementary material available at 10.1186/s12958-023-01140-3.

## Introduction

Preimplantation Genetic Testing (PGT) has been instrumental in preventing the transmission of genetic diseases within families. The PGT procedure is characterized by the biopsy of one or more cells from the developing embryo, followed by genetic testing to allow the transfer of non-at-risk embryos into the uterus.

PGT has evolved over the years: the practice of blastomere biopsy performed on cleavage-stage embryos (day 3) followed by either a fresh or a frozen-thawed embryo Transfer (FET) has shifted to trophectoderm biopsy on blastocysts (day 5/6) followed by vitrification of the blastocysts [1].

As embryo biopsy is invasive, safety concerns for the health of the offspring exist. One can hypothesize that the removal of embryonic cells has a detrimental effect on the development of the fetus and may affect the pregnancy course and, eventually, the pre- and postnatal growth of the offspring. It is well-known that, in the general population, birth size and inappropriate infant growth are associated with adverse health conditions later in life, including obesity and cardiovascular morbidity [2]. Therefore, it is necessary to investigate if babies born from biopsied embryos depict different or altered growth outcomes.

A few studies have evaluated the health of children born after PGT so far. Due to the relatively small sample sizes, the mix of types of biopsy (at cleavage-stage or blastocyst-stage) and the type of embryo transfer (frozen or fresh) in the available studies, strong conclusions regarding the impact on the offspring are lacking [3]. Furthermore, the available studies on blastocyst-stage biopsy are unfortunately mostly restricted to birth outcomes [4–8], resulting in a knowledge gap on growth and health beyond infancy. Also, the underlying reproductive background (infertility status) of the couples requesting PGT is rarely taken into account [6], despite its known impact on the health of the offspring [9].

Our group previously described outcomes at 2 years of age but in a modest group of children born after PGT using cleavage-stage biopsy [10]. The current study is more comprehensive and includes blastocyst-stage embryo biopsy; hence the results will reflect the recent changes in PGT practice with a shift towards trophectoderm biopsies.

This single-centre study aimed to investigate the impact of embryo biopsy on the health and growth of children up to 2 years of age, taking into account several parental and treatment factors.

## Materials and methods

### Study groups

All singleton deliveries following cleavage- and blastocyst-stage embryo biopsy followed by fresh or frozen-thawed embryo transfers between January 2014 and December 2018 were considered. This resulted in three embryo biopsy groups: cleavage-stage biopsy followed by vitrification on day 5 and frozen-thawed embryo transfer (embryo biopsy day 3, EBD3 FET), blastocyst-stage biopsy followed by vitrification on day 5 and frozen-thawed embryo transfer (embryo biopsy day 5/6, EBD5 FET) and cleavage-stage biopsy followed by fresh embryo transfer at day 5 (EBD3 FRESH).

The non-biopsy/control study population consisted of two groups of singleton deliveries after transferring a non-biopsied frozen-thawed (non-biopsy FET) and fresh blastocyst (non-biopsy FRESH) during the same study period.

Data are limited to single embryo transfer cycles. In both groups, embryos were obtained after ICSI, either with ejaculated or non-ejaculated, fresh or frozen sperm. Pregnancies after frozen embryo manipulation (FrEM), in vitro maturation of oocytes (IVM), oocyte vitrification and after oocyte/embryo donation were excluded.

Vitrification was the cryopreservation method used in all frozen-thawed embryo transfer cycles (biopsy and non-biopsy).

### PGT procedures

Patient counselling and genetic testing methods for PGT-M have been described previously [1]. Testing for monogenic diseases was either indirect, relying on haplotyping of genetic markers (STR/SNP) across the locus of interest and flanking regions, or direct, coupling pathogenic variant detection to genetic marker analysis. Chromosomal testing for PGT-SR/A has been performed by FISH or by WGA (Sureplex, Illumina) followed by either 24Sure array-CGH (Illumina) or by library preparation (KAPA HyperPlus, Roche), sequencing (Illumina) and an in-house-developed interpretation pipeline. Briefly, laser energy is used to open the zona pellucida on day 4 and then allow the embryo to grow to blastocyst. During the study period, the laser was used to remove cells (‘pulling method’). In the majority (84%) of the biopsies performed in cleavage-stage embryos, one cell was removed. Details on hormonal stimulation, oocyte collections, ICSI, embryo biopsy and transfer can be found in De Rycke et al. [11].

### Follow-up program

All children conceived in our centre and living in Belgium are eligible for follow-up. Depending on the mode of conception, all or random samples are approached for follow-up until young adulthood. In this case, all children born after embryo biopsy (study group) and living in Belgium are approached for follow-up. For the comparison group, a random group of children born after ICSI, either after fresh or frozen-thawed embryo transfer, and living in Belgium is approached. This strategy has been in place since 2004 due to the high numbers of ICSI children conceived in our centre. In practice, a computer program randomly selects 1 out of 3 families with children conceived after ICSI. Only these families are invited to our centre for an examination of their child in infancy and childhood. Pregnancy, delivery and birth data are provided by gynecologists and/or pediatricians or parents and are double-checked during the visit.

For all children (born after embryo biopsy or without embryo biopsy) in our follow-up program, a morphological assessment focusing on biometry and congenital malformations at 3–6 months, is performed by certified pediatricians and information on the course of infancy is added. The child’s second visit, which takes place at the age of around 2 years, focuses on growth but also includes information regarding psychomotor development, postnatal illnesses, hospital admissions, surgical interventions and medication intake.

Regarding congenital malformations, identical guidelines, definitions and classifications have been used for all children conceived in our centre since the introduction of our follow-up program for children born after ART [12]. A widely accepted definition of major anomalies, consisting of anomalies that generally cause functional impairment or require surgical correction is used.

### Ethical approval

All parents gave informed consent for participation in the follow-up program. The study was approved by the Ethics Committee of the UZ Brussel (B.U.N. 1432022000045).

### Study outcomes

Primary outcomes were anthropometric measures of weight, height and head circumference at birth, infancy (3-6 months) and childhood (2 years). Waist circumference and mid-upper arm circumference were additionally measured at childhood. The anthropometric measurements are expressed as standard deviation scores (SDS) to correct for gestational age and sex according to Belgian growth references [13]. Anthropometric outcomes at birth for gestational age <37 weeks were calculated using WHO growth charts [14]. Growth was expressed as gain (delta ∆) in weight and height: infancy weight gain was calculated as weight SDS at infancy minus birthweight SDS, and early childhood weight gain was calculated as weight SDS in early childhood minus weight SDS in infancy.

Neonatal outcomes explored were small-for-gestational age (SGA, <-1.28 SDS), large-for-gestational age (LGA, >+1.28 SDS), low birthweight (LBW; birthweight <2500g), preterm birth (gestation <37 weeks), macrosomia (birthweight >4000g), perinatal death and major congenital malformations.

### Statistical analysis

Descriptive statistics are presented as means ± standard deviation (SD) for continuous variables and as frequencies (percentages) for categorical variables. Statistical significance was set at the 5% level (*P*-value < 0.05).

Differences between the groups in anthropometric outcomes at birth, infancy and childhood were modelled using multiple linear regression. We modelled the relationship between the mode of conception (embryo biopsy or not) and each neonatal outcomes using logistic regression. Covariates for the final model were selected based on factors known from the literature to affect body size and/or were statistically different among the study groups. For reasons of uniformity, the same covariates were used in all models: treatment characteristics (number of oocytes at retrieval (<4, 4-18, >18)), maternal characteristics (nulliparity, age (<34, 34-40, >40 years), body mass index (<25, 25-30, >30), smoking, alcohol, gestational diabetes, pregnancy-induced hypertensive disorder).

The impact of embryo biopsy was assessed in frozen and fresh transfer cycles separately: blastocyst-stage embryo biopsy and cleavage-stage embryo biopsy followed by frozen-thawed embryo transfers were compared with outcomes after the transfer of non-biopsied vitrified blastocysts. Outcomes after cleavage-stage embryo biopsy followed by fresh embryo transfer were compared with outcomes after transfer of fresh non-biopsied blastocysts. As secondary and tertiary outcomes, we explored, within the PGT cycles, if the timing of biopsy (at day 3 or day 5/6) and if the vitrification process after embryo biopsy impacted children’s outcomes.

A subgroup analysis (biopsy versus non-biopsy groups) was performed in children born to parents with an infertility background. Three categories were distinguished: PGT for a genetic reason but no infertility in the couple, PGT for a non-genetic reason but infertility background (PGT-A in case of advanced maternal age, recurrent IVF failure or repeated miscarriages) or PGT for a genetic reason and infertility problem in the couple (due to a translocation in father and/or mother, congenital bilateral absence of the vas deferens, deletion on Y chromosome, fragile-X syndrome).

Statistical analyses were performed in Stata (version 17; Stata Corporation, College Station, TX, USA). 95% confidence intervals (95% CI) for the estimates are presented.

A power calculation (80% power, alpha of 0.05) demonstrated that the current sample was appropriate to detect a mean birthweight difference of 122g, 100g, and 104g for the comparisons of EBD5 FET vs non-Biopsy FET, EBD3 FET vs non-Biopsy FET, and EBD3 FRESH vs non-Biopsy FRESH, respectively.

## Results

### Study population and characteristics

**Fig. 1:**
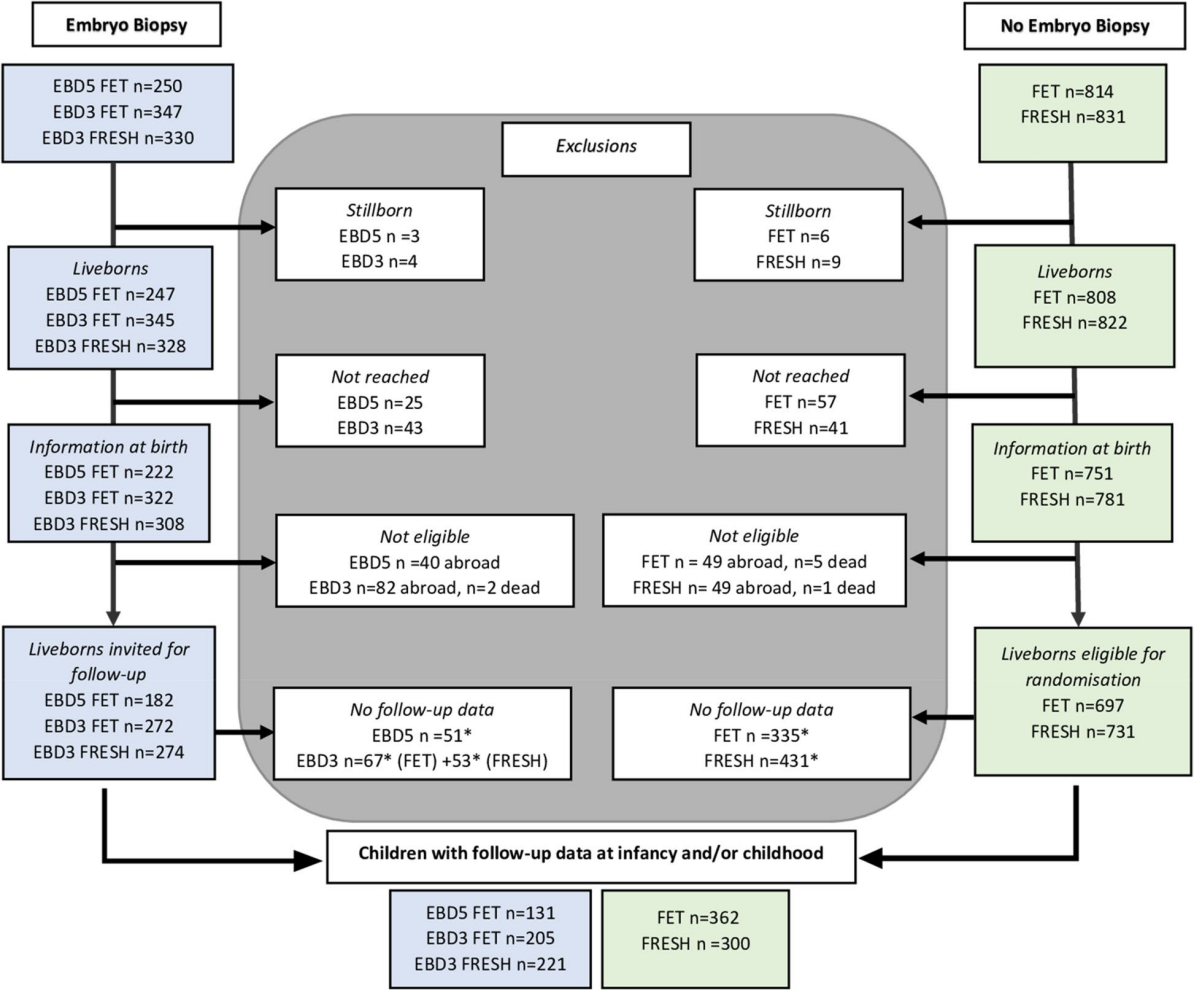
Flowchart showing participants in the embryo biopsy (EB) and non-biopsy groups. EBD5 (Embryo Biopsy Day 5), EBD3 (Embryo Biopsy Day 3), FRESH (fresh embryo transfer), FET (frozen-thawed embryo transfer). *included in non-participation analysis

All embryo transfers in the ‘embryo biopsy day 5/6 group’ (EBD5) were frozen-thawed (EBD5 FET) embryo transfers: 250 singleton deliveries resulting in 3 (1.2%) stillborn and 247 liveborn children (Figure 1). Birth data were available for 222 (88.8%) children and 182 were eligible for follow-up. Of these, 51 (38.9%) were not reached or refused to participate. Information on 131 children was available either at infancy and/or childhood. Follow-up data at the first visit were available for 126 (69.2%) children.

In the ‘embryo biopsy day 3 group’ (EBD3), either frozen-thawed (EBD3 FET) or fresh embryos (EBD3 FRESH) were transferred. In frozen-thawed embryo transfer cycles, 347 singleton deliveries resulted in 2 (0.6%) stillborn and 345 liveborn children. Birth data were available for 322 (92.7%) children and 272 were eligible for follow-up. Of these, 67 (24.6%) were not reached or refused participation. Information on 205 children was available either at infancy and/or childhood. Follow-up data at the first visit were available for 193 (71.0%) children. In fresh embryo transfer cycles, 330 singleton deliveries resulted in 2 (0.6%) stillborn and 328 liveborn children. Birth data were available for 308 (93.3%) children and 274 were eligible for follow-up. Of these, 53 (19.3%) were not reached or refused. Information on 221 children was available at infancy and/or childhood. Follow-up data at the first visit were available for 215 (78.5%) children.

In the non-biopsy FET group, 814 singleton deliveries resulted in 6 (0.7%) stillborn and 808 liveborn children. Of the 751 (92.2%) children with birth data recorded, 697 were eligible for follow-up. A random selection for follow-up resulted in data from 362 children either at infancy and/or childhood. Follow-up data at the first visit were available for 345 (49.5%) children.

In the non-biopsy FRESH group, 831 singleton deliveries resulted in 9 (1.1%) stillborn and 822 liveborn children. Birth data were collected for 781 (93.9%) children of which 731 were eligible for follow-up. A random selection for follow-up resulted in data from 300 children at infancy and/or childhood. Follow-up data at the first visit were available for 282 (38.5%) children.

Treatment and maternal characteristics of the biopsy and non-biopsy groups are presented in Table 1.

**Table 1:** Treatment and maternal characteristics of the biopsy and non-biopsy groups

	EBD5 FET *N=* 222	EBD3 FET *N=*322	EBD3 FRESH *N=*308	Non-biopsy FET *N=*751	Non-biopsy FRESH *N=*781
**Treatment characteristics**					
Number oocytes at retrieval (n, %)					
<4	7 (3.2)	3 (0.9)	10 (3.2)	3 (0.4)	20 (2.6)
4-18	162 (73.0)	191 (59.3)	254 (82.5)	469 (62.5)	679 (86.9)
>18	53 (23.9)	128 (39.8)	44 (14.3)	279 (37.2)	82 (10.5)
Supernumerary embryo(s) for vitrification					
Yes			182 (59)		654 (84)
No			126 (41)		127 (16)
Type of sperm (n, %)					
Ejaculated sperm	217 (97.7)	316 (98.1)	301 (97.7)	709 (94.4)	726 (93.0)
Non-ejaculated sperm	5 (2.3)	6 (1.9)	7 (2.3)	42 (5.6)	55 (7.0)
Origin of sperm (n, %)					
Partner sperm	220 (99.1)	314 (97.5)	303 (98.4)	644 (85.8)	674 (86.3)
Donor sperm	2 (0.9)	8 (2.5)	5 (1.6)	107 (14.2)	107 (13.7)
Indication for embryo biopsy (n, %)					
Genetic with infertility	53 (23.9)	18 (5.6)	35 (11.4)		
Genetic without infertility	133 (59.9)	302 (93.8)	267 (86.7)		
Non-genetic with infertility	36 (16.2)	2 (0.6)	6 (1.9)		
Indication for ICSI (n, %)					
Male factor				286 (38.1)	299 (38.3)
Female factor				160 (21.3)	142 (18.2)
Combined male + female factor				79 (10.5)	63 (8.1)
Idiopathic				226 (30.1)	277 (35.4)
Cycle protocol (n, %)					
Hormonal replacement therapy	109 (49.1)	158 (49.0)	0	279 (37.1)	0
(Modified) Natural cycle	106 (47.6)	154 (47.9)	0	458 (61.0)	5 (0.6)
Stimulated cycle	7 (3.1)	10 (3.1)	308 (100)	14 (1.9)	776 (99.4)
Cycle strategy (n, %)					
Freeze-all	222 (100)	228 (70.8)		396 (52.7)	
Previous embryo transfer	0 (0)	94 (29.2		355 (47.3)	
**Maternal characteristics**					
Maternal age at delivery, years (mean, SD)	34.2 (4.7)	31.7 (3.6)	31.8 (3.5)	32.9 (3.9)	31.8 (3.9)
Maternal BMI, kg/m^2^ (mean, SD)	23.5 (3.6)	23.9 (4.4)	24.3 (4.4)	23.5 (4.5)	23.8 (4.3)
Pregnancy-induced hypertensive disorder (n, %)	15 (6.8)	27 (8.4)	19 (6.2)	69 (9.2)	50 (6.4)
Gestational diabetes (n, %)	18 (8.1)	14 (4.3)	22 (7.1)	63 (8.4)	62 (7.9)
	*N=*197	*N=*274	*N=*252	*N=*698	*N=*674
Nulliparous (n, %)	126 (63.9)	160 (58.4)	160 (63.5)	222 (31.9)	156 (23.1)
	*N=*198	*N=*298	*N=*280	*N=*613	*N=*631
Maternal smoking (n, %)	1 (0.5)	6 (2.0)	7 (2.5)	21 (3.4)	27 (4.2)
	*N=*202	*N=*299	*N=*281	*N=*660	*N=*679
Maternal alcohol consumption (n, %)	9 (4.4)	20 (6.7)	20 (7.1)	44 (6.6)	52 (7.6)

*EBD5* Embryo Biopsy Day 5, *EBD3* Embryo Biopsy Day 3, *FRESH* fresh embryo transfer, *FET* frozen-thawed embryo transfer

A comparison of birth parameters, treatment and parental characteristics of the non-participants and participants is provided in Supplementary Table S1. Overall, participants and non-participants were comparable except for non-participating children in the EBD3 FET group who had a slightly higher birthweight SDS and mothers of non-participants in the non-biopsy FET group who were a few months older.

### Neonatal outcomes

In frozen-thawed embryo transfer cycles, the rates of premature birth, SGA, LGA, LBW and macrosomia were comparable between the biopsy (either at the blastocyst or cleavage stage) and non-biopsy group (Table 2), even after adjustment for confounders. Furthermore, a comparable proportion of children in the biopsy groups were admitted to the neonatal care unit (NCU) when compared to the non-biopsy groups: 8.7% in EBD5 FET and 9.6% in EBD3 FET versus 9.7% in Non-Biopsy FET; *P*=0.78 and *P*=0.99 respectively. Further, comparable rates of children were admitted to the NCU for >1 week (EBD5 FET 4.9%, *P*=0.62; EBD3 FET 5.3%, *P*=0.67; Non-Biopsy FET 6.2%).

**Table 2:** The impact of embryo biopsy on neonatal outcome in liveborn children

	EBD5 FET *N=*222	EBD3 FET *N=*322	EBD3 FRESH *N=*308	Non-biopsy FET *N=*751	Non-biopsy FRESH *N=*781	EBD5 FET vs Non-Biopsy FET	EBD3 FET vs Non-Biopsy FET	EBD3 FRESH vs Non-Biopsy FRESH
						Adjusted^a^ OR (95%CI)	Adjusted^a^ OR (95%CI)	Adjusted^a^ OR (95%CI)
Male sex	108 (48.6)	158 (49.1)	159 (51.6)	373 (49.7)	383 (49.0)			
Gestational age (weeks) (mean, SD)	39.0 (2.2)	39.2 (1.6)	38.9 (1.7)	39.1 (1.8)	38.9 (1.9)			
Premature birth <37weeks	15 (6.8)	26 (8.1)	23 (7.5)	69 (9.2)	57 (7.3)	1.00 [0.53, 1.88]	1.08 [0.65, 1.80]	1.08 [0.61, 1.91]
SGA <-1.28 SDS	5 (2.3)	7 (2.2)	14 (4.5)	21 (2.8)	33 (4.2)	1.16 [0.40, 3.40]	0.94 [0.36, 2.41]	1.44 [0.69, 3.00]
LGA >+1.28 SDS	8 (4.0)	13 (4.4)	8 (2.7)	29 (4.1)	10 (1.4)	0.91 [0.29, 1.21]	1.19 [0.57, 2.49]	2.17 [0.71, 6.54]
Low birth weight <2500g	12 (5.4)	14 (4.3)	21 (6.8)	42 (5.6)	53 (6.8)	1.02 [0.33,3.12]	0.87 [0.44, 1.73]	1.24 [0.70, 2.22]
Macrosomia >4000g	29 (13.1)	51 (15.8)	34 (11.0)	82 (10.9)	40 (5.1)	1.01 [0.60, 1.72]	1.42 [0.94, 2.15]	2.31 [1.35, 3.97]*
Major congenital malformations	5/204 (2.5)	13/301 (4.3)	13/292 (4.5)	23/687 (3.3)	26/713 (3.6)	0.93 [0.31, 2.80]	1.91 [0.86, 4.23]	1.22 [0.56, 2.65]

Values are expressed as number and percentage unless otherwise mentioned

*EBD5* Embryo Biopsy Day 5, *EBD3* Embryo Biopsy Day 3, *FRESH* fresh embryo transfer, *FET* frozen-thawed embryo transfer

^a^adjusted for neonatal (child’s sex), treatment (number of oocytes at pick-up), maternal characteristics (nulliparity, age, BMI, smoking, alcohol, pregnancy-induced hypertensive disorder, gestational diabetes)

*P<0.05

In fresh embryo transfer cycles, the rates of premature birth, SGA, LBW and LGA did not differ between the biopsy and non-biopsy group (Table 2), but macrosomia was more often found after embryo biopsy (AOR 2.31; 1.35-3.97). A comparable proportion of children were admitted to the NCU (11.9% in EBD3 FRESH versus 9.9% in Non-Biopsy FRESH; *P*=0.36). A similar proportion of children was admitted to the NCU for >1 week (EBD3 FRESH 6.2%, Non-Biopsy FRESH 6.2%; *P*=1.0).

The rate of major congenital malformations in liveborns following embryo biopsy was comparable to that in children born after the transfer of non-biopsied embryos for all comparisons (Table 2). Total major malformation rate, including malformations in stillborns and elective terminations, did not differ between the groups (EBD5 FET: 3.1%, EBD3 FET: 4.3%, EBD3 FRESH: 5.7%, Non-Biopsy FET: 4.8%, Non-Biopsy FRESH 4.0%; all *P*>0.05).

The timing of embryo biopsy (day 3 or day 5/6) did not affect neonatal outcomes (Supplementary Table S2). Likewise, vitrification after embryo biopsy did not impact the neonatal outcomes prematurity, LBW, LGA or macrosomia, except for an association with a lower SGA rate (AOR 0.34; 0.12-0.94) (Supplementary Table S2).

### Anthropometry at birth, infancy and childhood

In frozen-thawed embryo transfer cycles, birthweight, length and head circumference SDS did not differ between the biopsy (either blastocyst or cleavage stage) and non-biopsy groups. Adjustment for treatment and maternal confounders did not change the result (all *P* >0.05; Table 3). Weight, height and head circumference SDS in infancy and childhood were also comparable between the biopsy and non-biopsy groups. Likewise, weight and height gain from birth to infancy and infancy to early childhood were similar between the biopsy and non-biopsy groups (Table 3). No differences in waist and mid-upper arm circumference were noted between the biopsy and non-biopsy groups.

**Table 3:** The impact of embryo biopsy on anthropometrics from birth up to 2 years in frozen cycles

	EBD5 FET	EBD3 FET	Non-biopsy FET	EBD5 FET vs Non-biopsy FET		EBD3 FET vs Non-biopsy FET	
				Unadjusted mean difference (95% CI)	Adjusted^a^ mean difference (95% CI)	Unadjusted mean difference (95% CI)	Adjusted^a^ mean difference (95% CI)
**At birth**	*N=*222	*N=*322	*N=*751				
Weight (g)	3418 (583)	3434 (537)	3402 (532)	16 [-65.9,97.8]	-51.2 [-136.6, 34.2]	32 [-38.3,101.6]	13.6 [-59.6,86.8]
Weight SDS	0.17 (1.0)	0.14 (1.1)	0.11 (1.2)	0.06 [-0.09,0.21]	-0.05 [-0.20,0.12]	0.04 [-0.099,0.17]	0.03 [-0.12,0.17]
Length (cm)	50.5 (2.8)	50.5 (2ss.6)	50.3 (2.5)	0.18 [-0.22,0.59]	-0.08 [-0.51,0.35]	0.22 [-0.12,0.55]	0.12 [-0.24,0.48]
Length SDS	0.10 (1.3)	0.12 (1.3)	0.01 (1.2)	0.09 [-0.10,0.28]	-0.04 [-0.24,0.17]	0.11 [-0.047,0.28]	0.09 [-0.09,0.26]
Head circumference (cm)	34.6 (2.0)	34.7 (1.9)	34.5 (1.8)	0.12 [-0.20,0.44]	-0.09 [-0.43,0.26]	0.21 [-0.072,0.49]	0.11 [-0.19,0.41]
Head circumference SDS	0.02 (1.5)	0.08 (1.3)	-0.06 (1.4)	0.08 [-0.17,0.34]	-0.10 [-0.37,0.17]	0.15 [-0.070,0.36]	0.07 [-0.16,0.31]
**At infancy**	*N=*126	*N=*193	*N=*345				
Age (years)	0.4 (0.4)	0.5 (0.5)	0.5 (0.2)				
Weight SDS	0.16 (1.2)	0.05 (1.0)	0.16 (1.3)	0.01 [-0.25,0.26]	-0.02 [-0.30,0.26]	-0.10 [-0.31,0.11]	-0.17 [-0.40,0.06]
Height SDS	0.19 (1.3)	0.09 (1.1)	0.23 (1.4)	-0.04 [-0.31,0.24]	0.03 [-0.27,0.33]	-0.14 [-0.37,0.084]	-0.20 [-0.45,0.05]
Head circumference SDS	0.22 (1.1)	0.24 (0.9)	0.19 (1.1)	0.03 [-0.21,0.26]	0.01 [-0.25,0.26]	0.05 [-0.14,0.23]	-0.01 [-0.21,0.19]
**At childhood**	*N=*57	*N=*128	*N=*150				
Age (years)	2.3 (0.5)	2.2 (0.4)	2.2 (0.4)				
Weight SDS	-0.05 (0.8)	-0.03 (1.1)	-0.15 (1.0)	0.10 [-0.20,0.39]	0.18 [-0.16,0.52]	0.12 [-0.12,0.37]	0.03 [-0.25,0.31]
Height SDS	0.10 (1.1)	-0.17 (1.1)	-0.17 (1.1)	0.27 [-0.067,0.60]	0.52 [0.15,0.89]	-0.002 [-0.26,0.25]	-0.05 [-0.33,0.24]
Head circumference SDS	0.34 (1.1)	0.25 (0.9)	0.20 (1.0)	0.14 [-0.20,0.47]	0.11 [-0.27,0.48]	0.04 [-0.19,0.27]	0.02 [-0.25,0.29]
Waist circumference SDS	0.90 (0.8)	0.98 (1.0)	0.61 (1.1)	0.29 [-0.10,0.67]	0.36 [-0.08,0.80]	0.37 [0.07,0.66]	0.35 [0.03,0.69]
Mid-upper arm circumference SDS	0.27 (0.8)	0.46 (1.0)	0.19 (1.0)	0.08 [-0.27,0.10]	0.02 [-0.40,0.44]	0.27 [0.00,0.54]	0.28 [-0.03,0.59]
**Growth**							
∆ weight SDS birth to infancy	0.21 (1.1)	0.09 (1.1)	0.18 (1.3)	0.03 [-0.23,0.28]	0.12 [-0.16,0.41]	-0.09 [-0.30,0.13]	-0.04 [-0.27,0.19]
∆ height SDS birth to infancy	-0.13 (1.0)	-0.03 (1.0)	-0.22 (1.2)	-0.04 [-0.30,0.21]	0.05 [-0.23,0.33]	-0.21 [-0.43,0.02]	-0.23 [-0.47,0.07]
∆ weight SDS infancy to early childhood	0.18 (1.2)	0.01 (1.2)	0.22 (1.2)	0.08 [-0.28,0.44]	0.15 [-0.26,0.56]	0.19 [-0.08,0.45]	0.13 [-0.18,0.43]
∆ height SDS infancy to early childhood	-0.07 (1.1)	-0.23 (0.9)	-0.40 (1.5)	0.32 [-0.13,0.78]	0.46 [-0.06,0.97]	0.17 [-0.14,0.48]	0.17 [-0.19,0.52]

*EBD5* Embryo Biopsy Day 5, *EBD3 *Embryo Biopsy Day 3, *FET* frozen-thawed embryo transfer

^a^adjusted for treatment (number of oocytes at pick-up) and maternal characteristics (nulliparity, age, BMI, smoking, alcohol, pregnancy-induced hypertensive disorder, gestational diabetes)

In the fresh embryo transfer cycles, birth parameters and anthropometrics at infancy or childhood including waist and mid-upper arm circumference, were comparable in children born after embryo biopsy at day 3 and after the transfer of non-biopsied blastocysts. Also, weight and height gain were similar between the biopsied and non-biopsied groups. Adjustments for confounders did not change the result (Table 4).

**Table 4:** The impact of embryo biopsy on anthropometrics from birth up to 2 years in fresh cycles

	EBD3 FRESH	Non-biopsy FRESH	EBD3 FRESH vs Non-biopsy FRESH	
			Unadjusted mean difference (95% CI)	Adjusted^a^ mean difference (95% CI)
**At birth**	*N=*308	*N=*781		
Weight (g)	3304 (557)	3226 (539)	78 [5.58,149.6]	49 [-29.8,128.3]
Weight SDS	-0.15 (1.1)	-0.29 (1.0)	0.14 [0.002,0.28]	0.06 [0.092,0.21]
Length (cm)	49.8 (2.7)	49.6 (2.6)	0.24 [-0.11,0.60]	0.19 [-0.20,0.58]
Length SDS	-0.21 (1.2)	-0.32 (1.5)	0.11 [-0.05,0.28]	0.09 [-0.10,0.27]
Head circumference (cm)	34.3 (1.7)	34.1 (1.9)	0.20 [-0.09,0.49]	0.15 [-0.18,0.47]
Head circumference SDS	-0.24 (1.2)	-0.39 (1.5)	0.14 [-0.08,0.36]	0.09 [-0.15,0.34]
**At infancy**	*N=*215	*N=*282		
Age (years)	0.4 (0.4)	0.4 (0.2)		
Weight SDS	0.0 (1.1)	-0.03 (1.1)	0.03 [-0.17,0.23]	0.07 [-0.15,0.29]
Height SDS	0.03 (1.2)	-0.07 (1.2)	0.10 [-0.11,0.31]	0.09 [-0.14,0.32]
Head circumference SDS	0.02 (1.0)	0.02 (1.1)	0.00 [-0.18,0.18]	-0.01 [-0.21,0.20]
**At early childhood**	*N=*140	*N=*131		
Age (years)	2.1 (0.4)	2.2 (0.4)		
Weight SDS	-0.19 (1.2)	-0.20 (1.2)	0.01 [-0.27,0.29]	0.09 [-0.23,0.40]
Height SDS	-0.22 (1.2)	-0.18 (1.2)	-0.03 [-0.31,0.25]	-0.02 [-0.34,0.29]
Head circumference SDS	0.08 (1.0)	0.03 (0.9)	0.06 [-0.17,0.29]	0.05 [-0.21,0.31]
Waist circumference SDS	0.69 (1.2)	0.61 (1.1)	0.08 [-0.24,0.39]	-0.14[-0.22,0.50]
Mid-upper arm circumference SDS	0.04 (1.0)	0.36 (2.0)	-0.31 [-0.74,0.12]	-0.18 [-0.66,0.30]
**Growth**				
∆ weight SDS birth to infancy	0.22 (1.2)	0.42 (1.1)	-0.20 [-0.41,0.01]	-0.13 [-0.36,0.10]
∆ height SDS birth to infancy	-0.16 (0.8)	-0.09 (0.8)	-0.02 [-0.19,0.16]	-0.03 [-0.22,0.17]
∆ weight SDS infancy to early childhood	0.25 (1.1)	0.26 (0.9)	-0.07 [-0.27,0.13]	-0.03 [-0.25,0.19]
∆ height SDS infancy to early childhood	-0.27 (1.0)	-0.07 (0.9)	-0.20 [-0.44,0.03]	-0.19 [-0.45,0.07]

*EBD3* Embryo Biopsy Day 3, *FRESH* fresh embryo transfer,

^a^adjusted for treatment (number of oocytes at pick-up) and maternal characteristics (nulliparity, age, BMI, smoking, alcohol, pregnancy-induced hypertensive disorder, gestational diabetes)

The timing of embryo biopsy did affect neither anthropometrics at birth, infancy or childhood nor growth patterns until childhood (Supplementary Table S3). On the contrary, vitrification after embryo biopsy affected the outcomes: children born after embryo biopsy at cleavage stage followed by frozen-thawed embryo transfer had higher birth size (weight, height, head circumference SDS) when compared to children born after embryo biopsy at cleavage stage followed by fresh embryo transfer (Supplementary Table S3).

### Health outcomes in early childhood

There were no differences in the occurrence of mild and severe developmental (motor, language or social) disorders between the different biopsy and non-biopsy groups (all *P*>0.05; Table 5). The number of children with severe developmental problems was also comparable among groups. The number of children admitted to the hospital was comparable between the different groups; the main indication was infectious diseases in all groups (data not shown). Chronic (>3 weeks) medication use was also comparable between the groups and consisted mainly of inhaled corticosteroids. Finally, the number of children requiring a surgical intervention did not differ between the groups, except for the children in the EBD5 FET group who required fewer (8.8%) surgical interventions compared to their peers born after non-biopsied blastocyst transfer (21.3%; *P*=0.04).

**Table 5:** The impact of embryo biopsy on health outcomes at 2 years

	EBD5 FET *N=*57	EBD3 FET *N=*128	EBD3 FRESH *N=*140	Non-biopsy FET *N=*150	Non-biopsy FRESH *N=*131
Developmental disorder					
Mild	4 (7.0)	6 (4.7)	5 (3.6)	9 (6)	5 (3.8)
Severe	1 (1.7)	2 (1.6)	2 (1.4)	4 (2.6)	3 (2.3)
Hospital admission					
At least once	17 (29.8)	31 (24.2)	35 (25.0)	40 (26.6)	31 (23.6)
Chronic medication use					
Yes	6 (10.5)	14 (10.9)	13 (9.3)	17 (11.3)	14 (10.7)
Surgical intervention					
Yes	5 (8.8)*	20 (15.6)	18 (12.8)	32 (21.3)	18 (13.7)
For congenital malformation	1	6	5	5	3
Other reason	4	14	13	27	15

*EBD5* Embryo Biopsy Day 5, *EBD3* Embryo Biopsy Day 3, *FRESH* fresh embryo transfer, *FET* frozen-thawed embryo transfer

*P<0.05 for comparison between EBD5 FET vs Non-Biopsy FET; all other comparisons were not significantly different

### Subgroup analysis in children whose parents have an infertility diagnosis

In a sensitivity analysis comparing outcomes between children born after embryo biopsy to parents with an infertility background (*n*=150) and children born after the transfer of a non-biopsied embryo (*n*=1532), no differences were found for any of the anthropometrical measurements at birth, infancy or childhood in the adjusted analyses (Supplementary Table S4).

## Discussion

This single-centre cohort study compared anthropometrics, growth and general health of singletons born after embryo biopsy in frozen-thawed or fresh embryo transfer cycles with singletons born after transfer of non-biopsied embryos while adjusting for several treatment and parental characteristics. We found no impact of blastocyst- or cleavage-stage embryo biopsy in frozen-thawed and fresh embryo transfer cycles on growth and health in children until 2 years of age. Furthermore, the timing of the embryo biopsy (day 3 or day 5/6) did not affect anthropometrics at any age. However, within the embryo biopsy group, vitrification after embryo biopsy resulted in larger-sized babies at birth, but not thereafter. In our limited subgroup of children born to infertile parents, embryo biopsy did not affect anthropometry at any age.

The findings of this study on health outcomes at birth after cleavage-stage biopsy are overall in line with previous reports from our group [15] and others [16, 17]. Although not reported previously, we noted that children born after the transfer of fresh cleavage-stage biopsied embryos were more likely to be macrosomic than peers born after transfer of fresh non-biopsied embryos. Given this unexpected finding, we investigated if a higher cell stage at day 3 prior to embryo biopsy could explain the higher macrosomia rate. However, the mean number of cells at day 3 was comparable in the biopsied (9.02 ±0.12) and non-biopsied (9.05 ±0.07) groups. Furthermore, in the current study, we additionally reported on the effect of vitrification after cleavage-stage biopsy: children born after cleavage-stage biopsy followed by frozen-thawed embryo transfer were heavier and larger at birth and less likely to be born SGA. While these outcomes at birth following vitrification aligned with data from studies comparing outcomes in cycles without PGT [18], where higher mean birthweights and lower rates of SGA are described, it is not yet clear which factors are responsible for the observed differences [19]. In this study, we found a non-significant higher rate of LGA following vitrification and embryo biopsy, even though significantly higher rates have been repeatedly reported after frozen-thawed transfer of non-biopsied embryos [20, 21]. Reassuringly, our study did not find a negative impact of vitrification after embryo biopsy on anthropometrics and growth patterns beyond birth.

In parallel to the findings after cleavage-stage biopsy, our results showed no harmful effect of blastocyst-stage biopsy on anthropometrics at birth, infancy and childhood compared to outcomes after transfer of a non-biopsied blastocyst. This aligns with published data from China and the USA, where no adverse effects of blastocyst biopsy were reported on mean birthweight, rates of prematurity, LBW, SGA, LGA and macrosomia [4, 5, 7, 8, 22]. We further found a comparable prematurity rate, contrary to Li et al. (2021) [6] who reported a modest increased risk for prematurity in frozen embryo transfer cycles with PGT.

Given the shift towards blastocyst-stage biopsy rather than cleavage-stage embryo biopsy, it is reassuring that the timing of embryo biopsy does not negatively affect the health of the offspring. Given that the contemporary practice of PGT worldwide now involves blastocyst biopsy (for example, in our centre, blastocyst-stage biopsy was introduced in 2014 and currently accounts for nearly 90% of all biopsy cycles) more data regarding long-term health risks are expected to become available.

Information on congenital malformations after PGT is important but scarce and often gathered from questionnaires or interviews [4, 5, 23] or retrospectively retrieved from medical records [21] rather than based on dedicated examinations performed by trained clinicians [15, 24]. In this study, the rate of major congenital malformations in liveborns was similar for all comparisons of biopsied and non-biopsied groups. The rate of 2.5% congenital malformations in children born after transfer of a frozen-thawed biopsied blastocyst is similar to published data: 2.1% reported by Makhijani et al. [8] and 2.6% reported by He et al. [4] even though both rates were based on parental reported data. In addition, and unlike others, we aimed to provide a comprehensive estimate and to account for different approaches among countries regarding malformations detected during pregnancy, by presenting the total major malformation rate including malformations in stillborns and elective terminations. Reassuringly, these rates were also not different between biopsy and non-biopsy groups.

We additionally conducted a sensitivity analysis taking into account parental infertility status. When restricting the dataset to children born to parents with an infertility background, we did not observe any impact of embryo biopsy on anthropometrics and growth up to childhood. In a subgroup analysis of a large registry-based study of 14 285 cycles with a stated infertility diagnosis (female and/or male), an increased odds of preterm birth was observed in PGT cycles compared to non-PGT cycles [6]. However, this study did not provide data beyond the neonatal period. Studies stratifying according to infertility status in couples requiring PGT treatment are sparse in the literature. As such, our results, though based on a small sample size, are still informative but should be interpreted cautiously.

When comparing outcomes across studies, several methodological factors should be considered. Although more recent studies described neonatal outcomes after blastocyst-stage biopsy, most do not express birth parameters as standard deviation scores, SGA or LGA rates are not presented [4, 7, 8] or different definitions are used [5, 6]. In addition, specific information on treatment variables, such as cycle protocol [7] and covariates linked to maternal conditions during pregnancy, known to affect growth, such as gestational diabetes or hypertensive disorders, are often missing [6].

The current findings on health outcomes at 2 years of age were favorable and in line with our previous report describing children born after cleavage stage biopsy [10]. Indeed, all groups had similar hospital admission and surgical intervention rates and similar chronic medication use. Early childhood health outcomes in 390 children born after embryo biopsy have recently been reported [25]. That register-based study included all children born after PGT in Sweden between 1996 and 2019. But it is difficult to make comparisons or strong conclusions, since the described study population is rather heterogeneous, in terms of inclusion of IVF and ICSI cycles, vitrification and slow-freeze protocols, cleavage- and blastocyst-stage embryo biopsy and the number of embryos transferred.

One of the main strengths of this study is the inclusion of large and well-defined cohorts in terms of embryo biopsy (day 3 or day 5/6) and type of embryo transfer cycle (fresh or frozen) from a single centre and for which various treatment and parental characteristics were available. As such, our data add to the shortcomings in literature data as extensively described by Alteri et al. [3]. Moreover, laboratory practices for embryo biopsy and vitrification have been constant during our 5-year study period and in more than 85% of all cycles the same culture medium has been used. Furthermore, our results are based on specific clinical examinations performed by trained pediatricians, which is particularly important when assessing congenital malformations. Birth data were collected for over 92% of the children considered in this study. Furthermore, follow-up rates at infancy were reasonably high: growth data were available for nearly three-quarters (534/728) of the children born after embryo biopsy. In addition, most of the population characteristics of the non-participants were comparable to those of participants, which adds to the generalizability of the data and makes attrition bias less likely. Consequently, we explored longitudinal growth data expressed as weight and height gain, which is crucial as cross-sectional measurements at different ages might hide aberrant growth trajectories linked to adverse health outcomes later in life [26].

Our study also had some limitations. The number of children born after embryo biopsy to parents with an infertility diagnosis was limited as most of the PGT cycles in our centre were performed in patients with a genetic disorder without concomitant documented infertility. Therefore, the results from the sensitivity analyses should be interpreted cautiously. However, the main limitation of our study was the lack of prenatal growth data, which could have shed light on the findings of higher birth size in the group of children born after cleavage-stage embryo biopsy and frozen-thawed embryo transfer cycles compared with fresh embryo transfer cycles. Indeed, according to a large Nordic register study, the freeze-warming process was associated with excessive fetal growth from the third trimester of pregnancy onwards [27].

In conclusion, our study showed no adverse impact of embryo biopsy, either at the blastocyst or cleavage stage, on child growth and health up to 2 years of age. Although these findings were reassuring, longitudinal studies on long-term outcomes are recommended, as the implications of the current shift towards blastocyst biopsy in combination with freeze-warming protocols are yet to be identified.

### Supplementary Information

**Additional file 1: Supplementary Table S1.** Non-participation analysis. **Supplementary Table S2.** The impact of the timing of embryo biopsy and the vitrification process after embryo biopsy on neonatal outcomes. **Supplementary Table S3.** The impact of the timing of the embryo biopsy and the impact of the vitrification process after embryo biopsy on anthropometrics from birth up to 2 years. **Supplementary Table S4.** Sensitivity analysis: including children whose parents have an infertility background

## Data Availability

The data underlying this article will be shared on reasonable request to the corresponding author.
