# Supplementary material for: Cleavage-stage or blastocyst-stage embryo biopsy has no impact on growth and health in children up to 2 years of age
Source: Reprod Biol Endocrinol. 2023 Sep 22;21:87. doi: 10.1186/s12958-023-01140-3 (PMC10515414; doi:10.1186/s12958-023-01140-3)
Supplement: Supplementary file 1 — Additional file 1: Supplementary Table S1. Non-participation analysis. Supplementary Table S2. The impact of the timing of embryo biopsy and the vitrification process after embryo biopsy on neonatal outcomes. Supplementary Table S3. The impact of the timing of the embryo biopsy and the impact of the vitrification process after embryo biopsy on anthropometrics from birth up to 2 years. Supplementary Table S4. Sensitivity analysis: including children whose parents have an infertility background [file 12958_2023_1140_MOESM1_ESM.docx]

Supplementary Table S1. Non-participation analysis

|  | Non-participants | | | | | Participants versus non-participants | | | | |
| --- | --- | --- | --- | --- | --- | --- | --- | --- | --- | --- |
|  | EBD5 FET  N= 51 | EBD3 FET  N=67 | EBD3 FRESH  N=53 | Non-biopsy FET  N=335 | Non-biopsy FRESH  N=431 | EBD5 FET | EBD3 FET | EBD3 FRESH | Non-biopsy  FET | Non-biopsy  FRESH |
|  |  |  |  |  |  | P | P | P | P | P |
| **Treatment characteristics** |  |  |  |  |  |  |  |  |  |  |
| Number oocytes at retrieval (n, %)  <4  4-18  >18 | 3 (5.9)  33 (64.7)  15 (29.4) | 1 (1.5)  41 (61.2)  25 (37.3) | 3 (5.7)  44 (83.0)  6 (11.3) | 2 (0.6)  222 (66.3)  111 (33.1) | 10 (2.3)  378 (87.7)  43 (10.0) | 0.44 | 0.69 | 0.40 | 0.08 | 0.70 |
|  |  |  |  |  |  |  |  |  |  |  |
| **Maternal characteristics** |  |  |  |  |  |  |  |  |  |  |
| Maternal age at delivery, years (mean, SD) | 33.6 (4.8) | 30.9 (3.6) | 31.7 (3.7) | 33.1 (3.9) | 31.5 (4.1) | 0.9 | 0.41 | 0.68 | **0.008** | 0.36 |
| Pregnancy-induced hypertensive disorder (n, %) | 4 (7.8) | 4 (6.0) | 1 (1.9) | 25 (7.5) | 28 (6.5) | 0.76 | 0.79 | 0.21 | 0.15 | 1.00 |
| Gestational diabetes (n, %) | 2 (3.9) | 3 (4.5) | 2 (3.8) | 27 (8.1) | 41 (9.5) | 0.16 | 0.69 | 0.54 | 1.00 | 0.13 |
|  | N=37 | N=48 | N=33 | N=223 | N=303 |  |  |  |  |  |
| Maternal smoking (n, %) | 0 | 0 | 1 (3.0) | 11 (4.9) | 15 (5.0) | 1.00 | 1.00 | 0.51 | 0.16 | 0.55 |
|  | N=39 | N=50 | N=33 | N=257 | N=341 |  |  |  |  |  |
| Maternal alcohol consumption (n, %) | 0 | 5 (10.0) | 2 (6.1) | 22 (8.6) | 27 (7.9) | 0.12 | 0.55 | 1.00 | 0.15 | 1.00 |
|  |  |  |  |  |  |  |  |  |  |  |
| **Birth characteristics** |  |  |  |  |  |  |  |  |  |  |
| Gestational age (weeks) (mean, SD) | 38.8 (2.0) | 39.1 (1.5) | 38.9 (1.3) | 39.1 (1.5) | 39.0 (1.6) | 0.74 | 0.60 | 0.90 | 0.56 | 0.06 |
| Weight SDS (mean, SD) | -0.09 (0.9) | 0.38 (1.0) | -0.09 (1.0) | 0.13 (0.98) | -0.28 (1.0) | 0.07 | **0.03** | 0.97 | 0.72 | 0.59 |
| Height SDS (mean, SD) | -0.26 (1.1) | 0.21 (1.1) | -0.14 (0.97) | 0.07 (1.03) | -0.31 (1.17) | 0.09 | 0.43 | 0.76 | 0.37 | 0.33 |
| Admission to NCU (yes/no) | 4/42 (9.5) | 5/52 (9.6) | 3/45 (6.7) | 24/283 (8.5) | 29/376 (7.7) | 1.00 | 1.00 | 0.44 | 0.58 | 0.06 |

EBD5 (Embryo Biopsy Day 5), EBD3 (Embryo Biopsy Day 3), FRESH (fresh embryo transfer), FET (frozen-thawed embryo transfer)

Supplementary Table S2. The impact of the timing of embryo biopsy and the vitrification process after embryo biopsy on neonatal outcomes

|  | EBD5 FET vs EBD3 FET | EBD3 FET vs EBD3 FRESH |
| --- | --- | --- |
|  | Adjusted° OR (95% CI) | Adjusted° OR (95% CI) |
|  |  |  |
| Premature birth <37weeks | 0.65 [0.31, 1.38] | 0.96 [0.50, 1.84] |
| SGA <-1.28 SDS | 1.28 [0.34, 4.74] | 0.34 [0.12, 0.94]* |
| LGA >+1.28 SDS | 0.86 [0.33, 2.25] | 1.95 [0.76, 5.03] |
| Low birth weight <2500g | 1.28 [0.52, 3.11] | 0.48 [0.22, 1.05] |
| Macrosomia >4000g | 0.76 [0.43, 1.32] | 1.43 [0.86, 2.36] |
| Major congenital malformations | 0.40 [0.12, 1.33] | 1.00 [0.41, 2.40] |

EBD5 (Embryo Biopsy Day 5), EBD3 (Embryo Biopsy Day 3), FRESH (fresh embryo transfer), FET (frozen-thawed embryo transfer)

°adjusted for neonatal (child’s sex), treatment (number of oocytes at retrieval) and maternal characteristics (nulliparity, age, BMI, smoking, alcohol, pregnancy-induced hypertensive disorder, gestational diabetes)

*P<0.05

Supplementary Table S3. The impact of the timing of the embryo biopsy and the impact of the vitrification process after embryo biopsy on anthropometrics from birth up to 2 years

|  | EBD5 FET vs EBD3 FET | | EBD3 FET vs EBD3 FRESH | |
| --- | --- | --- | --- | --- |
|  | Unadjusted mean difference (95% CI) | Adjusted° mean difference (95% CI) | Unadjusted mean difference (95% CI) | Adjusted° mean difference (95% CI) |
| **At birth** |  |  |  |  |
| Weight (g) | -16 [-111.4,79.9] | -37 [-136.9,62.1] | 130 [43.9,215.6] | 139 [50.6,227.7] |
| Weight SDS | 0.03 [-0.15,0.20] | -0.01 [-0.20,0.18] | 0.29 [0.12,0.46]* | 0.30 [0.12,0.48]* |
| Length (cm) | -0.03 [-0.50,0.44] | -0.12 [-0.62,0.37] | 0.67 [0.24,1.09] | 0.76 [0.32,1.20] |
| Length SDS | -0.02 [-0.25,0.20] | -0.06 [-0.30,0.17] | 0.34 [0.14,0.54]* | 0.39 [0.18,0.59]* |
| Head circumference (cm) | -0.08 [-0.47,0.30] | -0.21 [-0.60,0.19] | 0.46[0.12,0.79] | 0.42 [0.08,0.77] |
| Head circumference SDS | -0.07 [-0.34,0.21] | -0.14 [-0.42,0.14] | 0.33 [0.10,0.56]* | 0.30 [0.06,0.54]* |
|  |  |  |  |  |
| **At infancy** |  |  |  |  |
| Weight SDS | 0.11 [-0.13,0.35] | 0.12 [-0.14,0.37] | 0.06 [-0.15,0.27] | 0.09 [-0.12,0.31] |
| Height SDS | 0.10 [-0.16,0.37] | 0.07 [-0.21,0.36] | 0.06 [-0.17,0.28] | 0.12 [-0.12,0.35] |
| Head circumference SDS | -0.02 [-0.24,0.20] | -0.08 [-0.31,0.15] | 0.22 [0.04,0.40]* | 0.24 [0.05,0.42]* |
|  |  |  |  |  |
| **At childhood** |  |  |  |  |
| Weight SDS | -0.03 [-0.34,0.29] | 0.01 [-0.34,0.37] | 0.16 [-0.12,0.44] | 0.20 [-0.09,0.50] |
| Height SDS | 0.27 [-0.07,0.61] | 0.34 [-0.04,0.72] | 0.04 [-0.23,0.32] | 0.13 [-0.17,0.43] |
| Head circumference SDS | 0.10 [-0.23,0.42] | 0.03 [-0.33,0.39] | 0.16 [-0.071,0.40] | 0.17 [-0.08,0.42] |
| Waist circumference SDS | -0.08 [-0.46,0.30] | -0.07 [-0.51,0.36] | 0.29 [-0.03,0.60] | 0.32 [-0.03,0.66] |
| Mid-upper arm circumference SDS | -0.19 [-0.56,0.18] | -0.37 [-0.79,0.05] | 0.42 [0.14,0.71]* | 0.32 [-0.05,0.68] |
|  |  |  |  |  |
| **Growth** |  |  |  |  |
| ∆ weight SDS birth to infancy | 0.11 [-0.13,0.36] | 0.16 [-0.10,0.42] | -0.12 [-0.35,0.10] | -0.12 [-0.35,0.12] |
| ∆ height SDS birth to infancy | 0.17 [-0.09,0.43] | 0.16 [-0.12,0.44] | -0.24 [-0.45,-0.02]* | -0.22 [-0.44,0.01] |
| ∆ weight SDS infancy to early childhood | -0.10 [-0.41,0.20] | -0.06 [-0.40,0.27] | 0.12 [-0.09,0.34] | 0.12 [-0.11,0.35] |
| ∆ height SDS infancy to early childhood | 0.16 [-0.15,0.46] | 0.21 [-0.12,0.53] | 0.04 [-0.18,0.27] | 0.05 [-0.19,0.29] |

EBD5 (Embryo Biopsy Day 5), EBD3 (Embryo Biopsy Day 3), FRESH (fresh embryo transfer), FET (frozen-thawed embryo transfer)

°adjusted for treatment (number of oocytes at retrieval) and maternal characteristics (nulliparity, age, BMI, smoking, alcohol, pregnancy-induced hypertensive disorder, gestational diabetes)

*P<0.05

Supplementary Table S4. Sensitivity analysis: including children whose parents have an infertility background

|  | EBD5 FET (n=89) + EBD3 FET (n=20) + EBD3 FRESH (n=41) vs Non-biopsy FET (n=751) + Non-biopsy FRESH (n=781) | |
| --- | --- | --- |
|  | Unadjusted mean difference (95% CI) | Adjusted° mean difference (95% CI) |
| **At birth** |  |  |
| Weight (g) | 88.6 [-3.34,180.6] | -25.9 [-122.4,70.6] |
| Weight SDS | 0.20 [0.02,0.38]* | -0.03 [-0.22,0.15] |
| Length (cm) | 0.18 [-0.26,0.63] | -0.17 [-0.65,0.30] |
| Length SDS | 0.07 [-0.14,0.29] | -0.09 [-0.32,0.13] |
| Head circumference (cm) | 0.34 [-0.01,0.69] | 0.09 [-0.28,0.47] |
| Head circumference SDS | 0.22 [-0.06,0.50] | 0.04 [-0.26,0.34] |
|  |  |  |
| **At infancy** |  |  |
| Weight SDS | 0.02 [-0.22,0.25] | -0.02 [-0.27,0.23] |
| Height SDS | -0.01 [-0.26,0.25] | -0.06 [-0.33,0.22] |
| Head circumference SDS | 0.02 [-0.21,0.24] | -0.03 [-0.27,0.21] |
|  |  |  |
| **At childhood** |  |  |
| Weight SDS | -0.21 [-0.51,0.10] | -0.23 [-0.56,0.11] |
| Height SDS | -0.24 [-0.55,0.08] | -0.25 [-0.60,0.10] |
| Head circumference SDS | 0.02 [-0.27,0.31] | -0.08 [-0.40,0.25] |
| Waist circumference SDS | 0.17 [-0.23,0.56] | 0.24 [-0.19,0.68] |
| Mid-upper arm circumference SDS | -0.40 [-0.90,0.10] | -0.48 [-1.04,0.08] |
|  |  |  |
| **Growth** |  |  |
| ∆ weight SDS birth to infancy | -0.07 [-0.32,0.18] | 0.05 [-0.22,0.31] |
| ∆ height SDS birth to infancy | -0.03 [-0.26,0.20] | -0.04 [-0.29,0.21] |
| ∆ weight SDS infancy to early childhood | -0.23 [-0.53,0.07] | -0.19 [-0.52,0.15] |
| ∆ height SDS infancy to early childhood | -0.16 [-0.52,0.21] | -0.10 [-0.51,0.31] |

EBD5 (Embryo Biopsy Day 5), EBD3 (Embryo Biopsy Day 3), FRESH (fresh embryo transfer), FET (frozen-thawed embryo transfer)

°adjusted for treatment (number of oocytes at retrieval, fresh or frozen transfer) and maternal characteristics (nulliparity, age, BMI, smoking, alcohol, pregnancy-induced hypertensive disorder, gestational diabetes)

*P<0.05
